# Snowmelt Runoff: A New Focus of Urban Nonpoint Source Pollution

**DOI:** 10.3390/ijerph9124333

**Published:** 2012-11-30

**Authors:** Hui Zhu, Yingying Xu, Baixing Yan, Jiunian Guan

**Affiliations:** 1 Key Laboratory of Wetland Ecology and Environment, Northeast Institute of Geography and Agroecology, Chinese Academy of Sciences, Changchun 130102, China; Email: zhuhui@neigae.ac.cn (H.Z.); yanbx@neigae.ac.cn (B.Y.); jn.a.guan@gmail.com (J.G.); 2 Graduate University of Chinese Academy of Sciences, Beijing 100039, China

**Keywords:** snowmelt runoff, urban, impervious surfaces, nonpoint source pollution, climate change

## Abstract

Irregular precipitation associated with global climate change had been causing various problems in urban regions. Besides the runoff due to rainfall in summer, the snowmelt runoff in early spring could also play an important role in deteriorating the water quality of the receiving waters. Due to global climate change, the snowfall has increased gradually in individual regions, and snowstorms occur more frequently, which leads to an enhancement of snowmelt runoff flow during the melting seasons. What is more, rivers just awaking from freezing cosntitute a frail ecosystem, with poor self-purification capacity, however, the urban snowmelt runoff could carry diverse pollutants accumulated during the winter, such as coal and/or gas combustion products, snowmelting agents, automotive exhaust and so on, which seriously threaten the receiving water quality. Nevertheless, most of the research focused on the rainfall runoff in rainy seasons, and the study on snowmelt runoff is still a neglected field in many countries and regions. In conclusion, due to the considerable water quantity and the worrisome water quality, snowmelt runoff in urban regions with large impervious surface areas should be listed among the important targets in urban nonpoint source pollution management and control.

## 1. Introduction

Urbanization has been the dominant trend of societal development all over the World during the last half century. Accompanying the economic growth and the accelerating urbanization, the threat of water issues such as water shortages, lack of the access to clean and safe water, *etc*., has become one of the major constraints on the development in many cities and regions [1,2,3,4]. Water shortages are reflected in not only the water quantity but also the water quality. However, the combined effects of point source (PS) and nonpoint source (NPS) pollution of the water environment make it difficult to fundamentally improve water quality [5,6]. Because of the increasing enforcement of PS pollution control and the difficulty to specifically identify or abate the NPS pollution, the proportion of NPS pollution in water contamination obviously increases and contributes to a large fraction of the water impairment.

In the majority of regions in the World, urban NPS pollution is the second important component of NPS pollution following agricultural NPS pollution [7]. In the USA, for instance, water quality in 13 percent of the rivers and streams, 18 percent of the lakes, ponds, and reservoirs, and 32 percent of the estuaries was considered impaired, mainly by urban NPS pollution [8].

The accelerating urbanization and the resulting increasing impervious surfaces are supposed to be the dominant driver for urban NPS pollution. Impervious surfaces prevent water from seeping into the ground and cause the occurrence of surface runoff. With the process of urbanization, the amount of impervious surfaces is increasing rapidly, thus more runoff is created and less water is able to sink in, or “infiltrate” into the ground. Water that travels slowly through the ground gets filtered by natural processes before it reaches the receiving water body, but water that travels too quickly to creeks and streams can pick up and carry a lot more sediment and other pollutants.

Usually, intense rainfalls are assumed to be the major flood-generating events in urban areas. However, flooding is also observed during snowmelt in urban environments in Scandinavia, Canada and the USA [9,10,11]. Flooding arising from snowmelts in early spring can also be observed in Northern China. Nevertheless, in contrast to the rainfall, snow is not mentioned in many urban NPS pollution study cases. That is probably because melt intensities are much lower than rain intensities, and because processes controlling snowmelt are purported to be the same in urban and rural basins. However, for snow, there are different processes and factors controlling runoff. Also, the snowmelt-related runoff conditions in a city are different in winter compared to storm runoff in summer [9]. On the other hand, the extreme weather associated with the global warming may lead more precipitation in some cold regions [12]. In other regions, the increasing temperatures may affect the length and time of the snow season and snow cover duration [13]. Accordingly, the load and characteristic of the snowmelt-related runoff may change obviously, which make it a major urban nonpoint pollution source and carrier.

Therefore, the aim of this study was to review and examine the urban NPS pollution problem considering both the rainfall and the snowmelt runoff, highlight the importance of the snowmelt runoff in urban NPS pollution against the background of global climate change, and to broach the foreground and development tendency of the snowmelt runoff research and management. The current work is important for both protecting and economizing the urban water resources and completing the theory of urban NPS pollution in cold regions.

## 2. Characteristics of Urban NPS Pollution

The research of urban NPS pollution began in the 1960s–1970s in developed countries, and subsequently a set of models and management systems based on the rainfall runoff characteristics and pollution load emerged. Storm water runoff discharge laws have been established in the USA, which requested that measures be taken during certain exploitation actions to control surface runoff, such as receiving water after storms being qualified [14]. In 2005, the management of urban NPS pollution was highlighted and the status of urban NPS pollution was changed from a local problem to a national problem in the US, marked by the decreeing of the US EPA government National Pollutant Discharge Elimination System (NPDES) program, which requires municipalities of a certain size to apply for permits to discharge storm water. The supporting documentation contains some assessment of the urban runoff pollution, including the results of field sampling, and proposes control measures. However, it was not until early the 1980s that urban NPS pollution was taken into consideration in China. The research work was firstly carried out in Beijing, and then in Shanghai, Hangzhou, Suzhou, *etc*. [15,16]. Limited cases can be found in Northeast China, except for a case study in Siping City [17].

Urban NPS pollution shares all the common characteristics of NPS pollution, however, obvious variations can be found between urban NPS pollution and the agricultural NPS pollution because of the difference in landform and surface features, land use patterns and anthropogenic activities [15]. The major characteristics of urban NPS pollution are reflected in the randomness of occurrence, the indefinite and extensive distribution, the complex processes, the hysteresis, the “first flush” effect for rainfall runoff (80 percent of the pollution volume is carried by 30 percent of the first runoff volume) [15,18], and the “first melt” effect for snowmelt runoff (the first action results in immediate runoff, usually involving small volumes of water and a minor portion of the annual pollution load, although concentrations may be high) [19], *etc*. For urban NPS pollution, the major pollutants include sediments, nutrients, heavy metals, oils and hydrocarbons, and oxygen-demanding substances, *etc*. [16]. Many factors can affect the characteristics and the load of urban NPS pollution, in which, the climate and hydrology, air quality, preceeding dry weather period, land use patterns, area of the impervious surfaces, urban drainage systems and urban sanitation all feature largely among the factors [20,21].

Although most of the studies pertaining to urban NPS pollution were focused on the runoff arising from big rainstorm events, there was tangible evidence that snowmelt runoff was responsible for a big fraction of urban environment contamination and the receiving water impairment. Thorolfsson and Brandt [11] studied urban storm runoff during summer and winter in Norway from 1988 through 1994 and found that snowmelt runoff had a much greater in volume than typically considered in drainage designs, resulting in much more winter flooding than that in summer. Besides, an urban storm-runoff model that considers snowmelt and rainfall was also produced [11], but it was concluded that there was still a notable lack of experience about urban storm runoff during the winter season. On the other hand, despite previous conclusions that not all kinds of pollutants were prone to dissolve in snowmelt [22], snow has been shown to store high levels of various pollutants including lead, hydrocarbons, polychlorinated biphenyls, sulfate and other metals and solids [23,24]. For instance, Blecken *et al*. [25] found that due to the change of runoff intensity and high sediment loads in the snowmelt runoff, snowmelt runoff can cause an increased proportion of fine-grained sediment fractions (<0.063 mm) in spring, and the retention of metals can also be observed because of the low turbulence in the water and the presence of organic material,. Thus the snowmelt runoff indeed must be taken into consideration in studying and controlling the urban NPS pollution. However, it is difficult to identify and control the urban snowmelt runoff due to the anthropogenic redistribution activities, the complex melting process, the variety of runoff condition in cities and the global climate change.

## 3. Models of Urban NPS Pollution

Models play critical roles in urban NPS pollution research. On-site measurement and monitoring of urban NPS pollution is expensive and time consuming. Runoff events are intermittent, and long-term records would be required to measure the runoff from a specific site. In addition, there are many complicating factors affecting urban NPS pollution. For these reasons, models are, in most cases, the only reasonable tools for runoff prediction, assessment, and management. Earlier models for urban NPS pollution were mostly performed based on the river quality change arising from land utilization. Most of them belong to the statistic models class. The models mainly focus on the rainfall runoff characteristics, individual rainstorms and long-term runoff pollution load, and the relationship between runoff amount and the land utilization. The most famous models include the Storm Water Management Model (SWMM), the urban surface runoff model (STORM), the Wallingford model and the MOUSE model.

The SWMM was first developed back in 1971 by the US EPA and has undergone several major upgrades. The model is a dynamic rainfall-runoff simulation model used for single event or long-term simulation of runoff quantity and quality from primarily urban areas. It continues to be widely used throughout the World for planning, analysis and design related to storm water runoff, combined sewers, sanitary sewers, and other drainage systems in urban areas, with many applications in non-urban areas as well [26]. The STORM model, developed in 1973, is designed to model urban watersheds and is capable of calculating loads and concentrations of water quality parameters, such as suspended and settleable solids, biochemical oxygen demand, total nitrogen, orthophosphate, and total coliforms, besides, it is also capable of calculating land surface erosion. STORM requires hourly precipitation data to model the stormwater elements which result in a difficulty of collecting the vast basic data needed. The Wallingford Model was explored in 1978 by the Institute of Hydraulics, Wallingford, U.K. The model can simulate a storm and waste water system and was widely used in the operation, design and layout of storm equipment. The MOUSE model developed by DHI was a synthetic model which can simulate the hydrology, the hydraulic and the water quality of urban sewerage and drainage systems [27]. Besides the above classical models, many new models based on rich experience appeared in the 1980s–1990s. The new models tend to focus on the management and risk assessment of NPS pollution, which greatly promoted urban NPS pollution model research. With the development of computer and 3S techniques, models with powerful functions, such as special data management, database technique and digital expression were explored based on drainage scale, providing advantages to urban NPS pollution research.

The importance of snowmelt runoff in melting seasons emerged gradually in the recent decades, especially in cold regions, thus some urban NPS pollution models were explored specially for the snowmelt runoff events. One of the most typical models is the Urban Snow Model (USM) built by Valeo and Ho in 2004 [28]. USM uses an energy balance scheme at an hourly time step, changes in urban snow albedo, and incorporates eight different types of redistributed snow cover. The full energy budget version of USM outperformed all other models in terms of time to peak, peak flow rate and model efficiency. Its modified version is recommended when a lack of data exists.

Conceptual snowmelt runoff models have proven useful on estimating discharge from remote mountain areas, including the various spanning ranges of the Himalayas. Such models can provide fairly accurate predictions of water availability for operational purposes (e.g., irrigation and hydropower). However, the capability of these models to address characteristic components of water disputes such as diversions, storage and withholding is limited. Contemporary disputes between India and Pakistan are partly due to the snowmelt-derived water resources from the Upper Indus Basin, and water balance accounting methods need to be improved to cope with this issue. Kult *et al*. [29] presented a research agenda focused on providing refined hydrological contributions to solve water disputes. The snowmelt runoff model built by Tahir *et al*. [30] in 2011 can be used efficiently in the snow- and glacier-fed sub-catchments of the Upper Indus River Basin (UIB). The application of the snowmelt runoff model under future climate (mean temperature, precipitation and snow cover) change scenarios indicated a doubling of summer runoff until the middle of this century.

However, for most countries and regions, especially the countries where urban NPS pollution research is in an infancy status, the models fail to be applied suitably. For instance, most of the models concerning surface runoff have been widely applied in China during the last few years, but the urban development mode and basic conditions in China vary largely from those of cities in other countries. The comparatively high building mass and fewer greenbelt zones result in more impervious floor area in the cities of China. On the other hand, the poor urban sanitation conditions, and the lack of basic data lead to the failure to use the models suitably. To date, there is neither a good model nor perfect experience on management and control of urban surface runoff in China [14], let alone models or methods capable of comprehensively considering both the rainstorms in summer and the snowmelt runoff in winter and early spring.

## 4. Snowmelt Runoff under the Global Warming

### 4.1. Water Quantity of Snowmelt Runoff

There is no dispute about the global climate changes induced by greenhouse gases, which are likely to have important repercussions on the water cycle and water resources in general. Snow is the most sensitive component of the water cycle under climate change conditions [13]. The response of snowmelt in cold regions to the climate change is mainly reflected in the amount of snowfall, the frequency of snowstorm events and the variety of melting processes.

Urban flooding in cold regions, which is dominated by both rainfall and snowmelt, is especially vulnerable to climate change. With the increasing global temperature, the precipitation in some regions has presented a slowly increasing trend. In general, an increase of the mean precipitation between 30°N and 70°N has been observed, and this is also true for the area between 0°S and 70°S southern latitude. In addition to these global changes, some regional changes have been observed. In North America, the northern part of Canada and Alaska, and southern part of Canada, increasing precipitation to a different extent was observed during the last few decades [12]. The precipitation calculation includes both the summer rainfall and the winter snowfall, and that means people in the corresponding regions should concentrate on the amount of runoff both in summer and in winter. For the past four winters, persistent above-normal snow cover was found in much of the northern US, East Asia and Europe [31]. Besides the increase of the snowfall amounts, the frequency of snowstorm events is likely to increase too. In 2007, the Intergovernmental Panel on Climate Change (IPCC) documented that extreme weather events have become more common globally, including heavy rainfall and snowfall events, intense droughts, heat waves, and tropical cyclones [32]. Heavy snowfall requires moist air which results from warm conditions that evaporate water from the ocean or large lakes. When the moist air travels over land and meets colder air, the moisture will freeze and fall as snow. If the air over the ocean or large lakes becomes warmer, it will hold more moisture and the snowfall will increase. Thus, along with the global climate change, the higher evaporation rates may be responsible for the frequent heavy snowfall events.

In cold regions a change in ambient air temperature has the potential to trigger a discontinuous response in runoff by changing the length and timing of the snowmelt season [33]. Snowmelt in urban area is considered to be dominated by net radiation fluxes, sensible heat flux, turbulent exchanges and heat exchange at the snow-soil interface, *etc*. [28]. The global climate change and the resulting increasing temperature are prone to affect the above factors directly or indirectly, leading to changes in the snowmelt process and the length and timing of the snowmelt season. Arnell [34] simulated large scale European water resources on the basis of GCM predictions for 2050 and identified areas with winter snow cover as being particularly sensitive to climate change. Northern Norway and southern Sweden showed an increase in winter runoff at the expense of spring peak flow, while eastern Sweden showed spring flow peaks occurring a month earlier. In addition, for rain-on-snow events which mainly happen under warmer temperature conditions, the frozen soils in cities contribute to considerable runoff increases, and can be greater than in summer storms [28].

### 4.2. Water Quality of Snowmelt Runoff

If runoff response to precipitation in cities is the engine to trigger urban NPS pollution, the content and solubility of the chemicals in the runoff fuel the engine. Therefore, runoff quality much attention should also be paid to better understanding the urban NPS pollution. Four potential factors result in the worrying quality of runoff in melting seasons:

(1) When it snows, atmospheric pollutants can be absorbed by the snow, eventually contributing to the runoff water contamination. In contrast to summer, the air quality in winter is usually much worse than that of other seasons because of the combustion of coal for heating. Here, Changchun City, the capital city of Jilin Province, Northeast China was chosen as an example. It is at 124°18'–127°02' east longitude, 43°05'–45°15' north latitude, with an urban area of 3,583 km^2^ and a population of 7.057 million [35]. With an average temperature of 5.2 °C and an average annual rainfall of 522–615 mm, it is a typical northern city in China, which combusts coal for warming in winter. The ambient air quality data of Changchun City was downloaded from the website of the China Meteorological Data Sharing Service System. Figure 1 shows the monthly air pollution index of Changchun City from 2007 to 2009. The main objective of the air pollution index (*API*) is to measure the air quality with respect to its effects on the human health. Typically this is a daily quality index. A daily *API* has been proposed by Environmental Protection Agency (EPA). It is deﬁned with respect to the ﬁve main common pollutants: carbon monoxide (CO), nitrogen dioxide (NO_2_), ozone (O_3_), particulate matter (PM_10_) and sulphur dioxide (SO_2_). The evaluation of the *API_s,p_* at station *s* for pollutant *p* (*API_s,p_*) is carried out by a linear interpolation of the reference scale values:

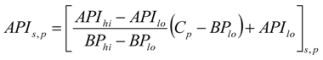
(1)
where *API_s,p_* is the value of the *API* at site *s* for pollutant *p*; *C_p_* the daily reference concentration of pollutant *p*; *BP_hi_* the lowest breakpoint of pollutant *p* that is greater than or equal to *C_p_*; *BP_lo_* the highest breakpoint of pollutant *p* that is less than or equal to *C_p_*; *API_hi_* the *API* value corresponding to *BP_hi_* of pollutant *p* and *API_lo_* the *API* value corresponding to *BP_lo_* of pollutant *p* [36]. As shown in Figure 1, the pollution indexes in winter seasons (November–March) are much higher than that in other seasons, indicating a worse air condition of snow than that of rain. In addition, the longer antecedent dry weather period in winter may also enhance the runoff pollution level.
  **Figure 1:**
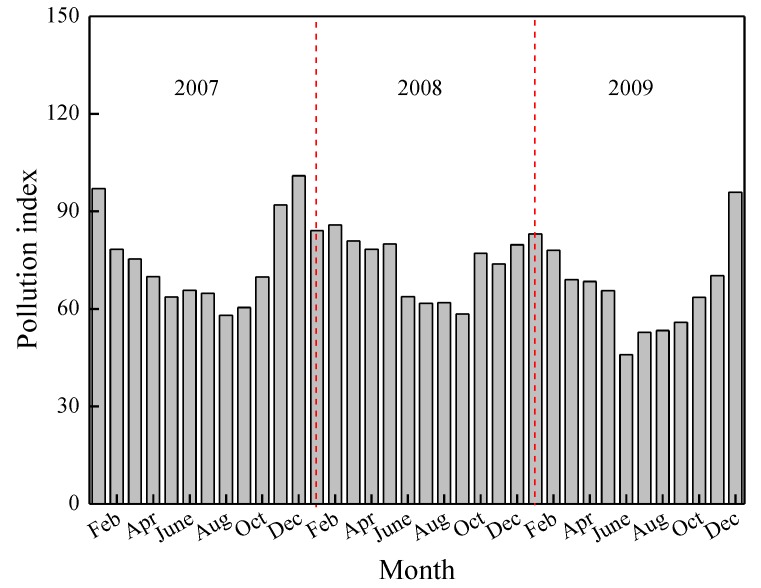
Monthly variation of air pollution index of Changchun City in 2007–2009.
(2) Dust and other pollutants on the impervious surface in cities are important components in snowmelt accompanying the process of runoff scouring. However, unlike the runoff in summer, the cold weather in winter hinders the melting process of snow, resulting in the accumulation of snow cover, and the runoff will not happen until the temperature increase to a suitable level. Therefore, with the accumulation of snow, the contaminants accumulate due to the pollutants on impervious surfaces and dropping from the upper atmosphere. In a word, the accumulation effects may deteriorate the runoff quality to some extent. Slow accumulation and fast transportation of pollutants into water can make pollutant loads rocket within a short period. However, rivers awaking from freezing are frail ecosystems with poorer self-purification capacity due to the lower temperature and lower microorganism activity, which seriously threatens the receiving water quality. For example, the suspended solids, permanganate index and nitrate concentration observed in April was about 2–4 times higher than that of January and February in the Dongdaqiao section of Yitong River, which is the most important receiving river of Changchun City. In addition, the conductivity presented the same trend [37].
(3) Applying snowmelt agents is one of the most common measures used to tackle snow on roads and highways in most of the cold regions of the World. Furthermore, frequent big snowstorm events these years may increase the amounts of snowmelt agent applied. When deicers are applied or the sun shines on heat absorbing paved areas, it results in a winter-long sequence of chemically-driven melt events in which saline water carries accumulated road pollutants into drainage systems and local receiving waters [19]. As a result, the snowmelt agent becomes a unique type of pollutant in snowmelt runoff. Though there are a variety of snowmelt agents, the major and common component of snowmelt agent is inorganic chloride. Thus, besides the large amount of metallic cation which may increase the conductivity and salinity of the receiving waters, the chlorine deicer has obvious adverse effect on concrete brick, and could reduce the pH value of water [38]. Road salt deicers, especially NaCl and CaCl_2_, are used extensively in the US and elsewhere. The results demonstrated addition of NaCl, and especially 5 g/L CaCl_2_·H_2_O, resulted in stimulated anaerobic production of dissolved Mn(II) and Fe(II) in the sediment pore waters. This is hypothesized to be the result of nutrient release (e.g., K, Ca, trace elements) via an ion exchange processes, which fuels primary production and subsequent organic matter degradation in the cores treated with NaCl or CaCl_2_ [39]. Therefore, the snowmelt agent in snowmelt runoff should be taken into consideration in any research and control of the runoff in melting seasons.
(4) Compared with summer, the ecological purification function of greenbelts in winter is much lower, which leads to worse water quality. According to Westerlund’s study in northern Sweden [40], the concentrations during the melt period were significantly higher compared to those during the rain period for all particle size intervals. During the melt period, it had about a five times higher number of particles, for all particle sizes, than the rain period, furthermore, investigated particle sizes and total suspended solids were highly correlated with total concentrations of Cd, Cu, Ni, Pb, and Zn. The highest correlations were found for total suspended solids and particle sizes 6–9 μm. According to other researchers, removal rates of TSS, COD, BOD, TP, TN, Zn, and Fe in runoff can reach above 60 percent after the runoff flows through vegetated areas [41], and this can be attributed to the physical interception, the absorption of plants, and the microbial decomposition, *etc*. [42]. The ability of greenroofs to reduce problems of urban stormwater runoff quantity [43] and quality [44] are being investigated more and more intensively. Teemusk and Mander [45] analyzed the chemistry characters of the runoff samples near the city centre of Tartu, Estonia, the results showed that the slower the runoff rate, the higher the concentrations of total N, NH_4_-N and organic material (after BOD_7_ and COD) in the runoff water. Total P concentration did not vary significantly in relation to water discharge. Heavy rain washed more phosphates and also nitrates out of the greenroof. In snow melting water, the concentrations of all components were greater on the greenroof due to the accumulation of atmospheric pollutants in snow. As the measurements showed, the greenroof runoff always contained more sulphates and Ca-Mg-salt. On the other hand, for example, the concentrations of P and N, and also COD and BOD_7_, were higher in the runoff water of the reference roof in the case of moderate runoff. However, in winter, the only residual function of the greenbelt may be the physical intercepting ability of some wizened plants. As for the biological plant absorption and the microbial decomposition function, it cannot be expected to be taken into consideration. Thus, the low natural purification ability of greenbelts in urban area leads to higher risk of water impairment occurring during melting seasons.

## 5. Management and Control Strategy of Urban NPS Pollution

**Figure 2:**
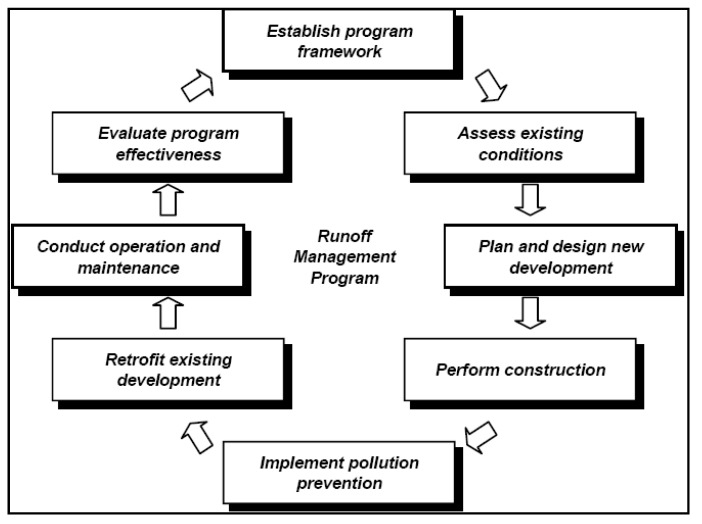
Components of comprehensive runoff management program [39].

The general method of controlling urban NPS is to apply best management practices (BMPs) which has been used by many projects in many countries [46]. BMPs was defined by US EPA as any methods, practices and operation programs of mitigation and precaution in water resources pollution including the operation and maintenance program of engineering and non-engineering practices [47]. In practical application, it is adopted rationally according to local weather and natural geography conditions in different areas [14]. Besides, developed countries have built many systemic management methods for urban NPS pollution. The US EPA [48] documented a comprehensive runoff management program (Figure 2) to provide technical assistance to state and local program managers and other practitioners on the best available, most economically achievable means of managing urban runoff and reducing NPS pollution of surface and ground waters from urban sources. In most developing countries, however, the management and control of urban NPS pollution is not optimistic due to the long-term neglect of urban NPS pollution issues. In China, for instance, there is neither a policy with respect to urban NPS pollution nor laws pertaining to control of urban NPS pollution. This is not conducive to control of urban NPS pollution. Thus, the policy-making may become one of the most urgent and necessary works for controlling urban NPS pollution in developing countries. The contents of policies should include the management, precaution, the monitoring, and the rewards and punishment systems. In view of the potential risk of snowmelt runoff under the climate change, the policy should also consider both the summer flooding and the snowmelt runoff in cold regions. Local governments at all levels should also work out local policy guidance on the storm and sewage sewers design in accordance with actual conditions.

In addition to the management system, exploring the ecological purification technique is recommended for tackling urban NPS pollutants. The constructed wetlands and grass greenbelts are the preferred tools of ecological purification. Constructed wetlands have gained international interest and applications due to their low maintenance and operational costs, and their high removal capacity for many kinds of pollutants [49,50]. For instance, in the U.K., a significant amount of money has been spent to investigate reed bed schemes in England and Wales [51] to purify the water. In Sweetwater, USA, a constructed wetland was applied for treating urban runoff and it does reduce the amount of nutrients and metals in the water column [51]. In China, Liu *et al*. [52,53] obtained good purification ability for the NPS pollutant nitrogen with constructed wetlands. Zhang [36] used a grass swale system for purifying urban storm runoff with satisfactory results. In New Delhi, India, constructed wetlands are also applied for urban runoff control [54]. However, in general, the application of ecological purification technique in urban NPS pollution control in developing countries has lagged behind that in developed countries due to the lack of economic support and the needed technical expertise. More cases of practical application are needed, but not always stay on the experimental scale. In addition, in view of the considerable snowmelt runoff, the lower microorganism activity under cold conditions and the resulting lower purification capacity, exploring zoology purification techniques which have the capacity to be operated under cold environments is important and of far-reaching significance in this field.

## 6. Conclusions

In the current review study, progress of NPS pollution studies considering both the summer flooding and the snowmelt runoff was examined. The snowmelt runoff was highlighted for its more and more important status in urban NPS pollution under the conditions of global climate change. With the global temperature increase, the considerable snowmelt quantity and the worrisome water quality in cold regions make it urgent to take measures to manage snowmelt runoff in melting seasons despite of the lack of data in this field now. Policy-making and application of ecological treatment techniques are recommended. The next step of the study is to apply the runoff model to simulate and forecast the summer stream flow as well as to evaluate the impact of future climate scenarios.
